# Plant-Derived Compounds Targeting Pancreatic Beta Cells for the Treatment of Diabetes

**DOI:** 10.1155/2015/629863

**Published:** 2015-10-26

**Authors:** Yoon Sin Oh

**Affiliations:** ^1^Lee Gil Ya Cancer and Diabetes Institute, Gachon University, Incheon 406-840, Republic of Korea; ^2^Gachon Medical Research Institute, Gil Hospital, Incheon 405-760, Republic of Korea

## Abstract

Diabetes is a global health problem and a national economic burden. Although several antidiabetic drugs are available, the need for novel therapeutic agents with improved efficacy and few side effects remains. Drugs derived from natural compounds are more attractive than synthetic drugs because of their diversity and minimal side effects. This review summarizes the most relevant effects of various plant-derived natural compounds on the functionality of pancreatic beta cells. Published data suggest that natural compounds directly enhance insulin secretion, prevent pancreatic beta cell apoptosis, and modulate pancreatic beta cell differentiation and proliferation. It is essential to continuously investigate natural compounds as sources of novel pharmaceuticals. Therefore, more studies into these compounds' mechanisms of action are warranted for their development as potential anti-diabetics.

## 1. Introduction

The prevalence of diabetes and metabolic disease is rapidly increasing worldwide and is becoming a major health problem [1]. Diabetes affected an estimated 285 million people worldwide in 2013 and is expected to affect 439 million people by 2030 [2]. Over 90% of diabetic patients have type 2 diabetes and the cost of care is a large economic burden for many countries. Indeed, the estimated costs of diabetes care in the USA in 2012 were 245 billion US dollars, which was a 41% increase from the 2007 estimate ($174 billion) [3].

**Figure 1:**
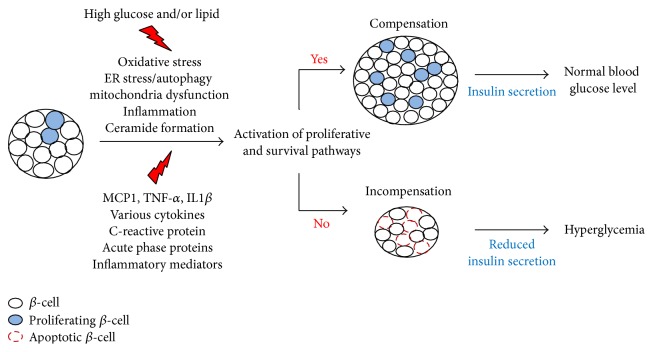
Mechanisms underlying pancreatic beta cell failure in type 2 diabetes. In type 2 diabetes, progressive pancreatic beta cell loss can be caused by environmental factors such as lipids (lipotoxicity), glucose (glucotoxicity), and inflammatory mediators secreted by the adipose tissue. Decompensation of pancreatic beta cell mass induces pancreatic beta cell apoptosis and decreases insulin secretion, thereby accelerating the hyperglycemic state.

Diabetes is characterized by hyperglycemia, which can cause diabetic complications including cardiovascular disease, nephropathy, retinopathy, and neuropathy [4]. Disturbance of glucose homeostasis is a major factor in the development of hyperglycemia. Insulin released by pancreatic beta cells is the key hormone responsible for glucose metabolism homeostasis [5]. In both type 1 and type 2 diabetes, absolute or relative insulin deficiency results in the development of hyperglycemia [6, 7]. In type 1 diabetes, pancreatic beta cells are damaged by immunological factors, such as cytokines and macrophages or T cells activated by autoimmune responses. Type 2 diabetes results from both insulin resistance and relative insulin deficiency that cannot compensate for the insulin resistance. In type 2 diabetes, pancreatic beta cells are damaged or become dysfunctional because of the persistently high glucose or lipid levels, inflammatory mediators released from the adipose tissue and endoplasmic reticulum, or oxidative stress (Figure 1). Thus, maintaining pancreatic beta cell function may be a strategical approach for the prevention and treatment of diabetes. Research of novel and cost-effective agents that can enhance pancreatic beta cell function or can increase pancreatic beta cell mass is important for the discovery of novel antidiabetics.

**Figure 2:**
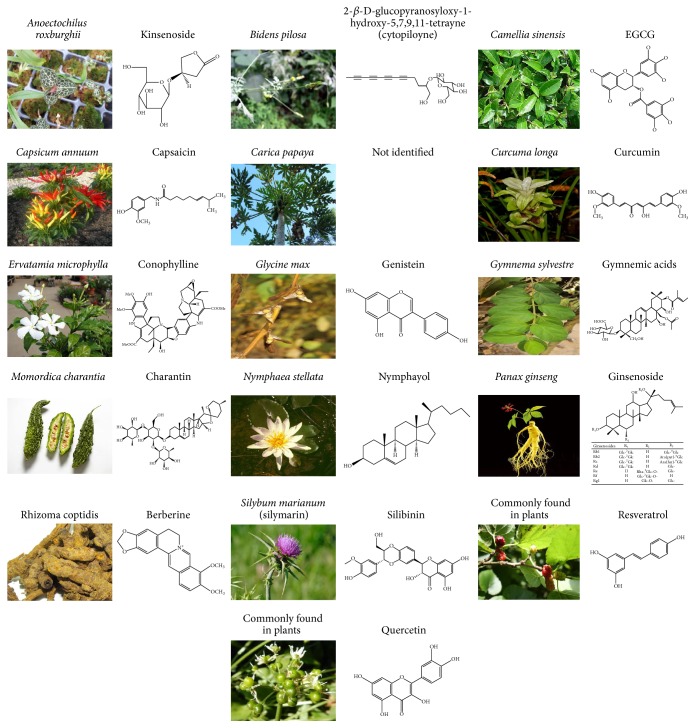
Structural features of plants and bioactive compounds that affect pancreatic beta cell function and diabetes.

The chemical compounds/substances found in living organisms are known as natural compounds. The various sources of these natural compounds include plants, animals, and microorganisms [8]. Natural bioactive compounds are a source of novel pharmaceuticals because of their diversity, which enables the synthesis of drugs that differ from other chemical compounds in terms of their complex structures and biological potency [9]. About 50% of the drugs approved by the US Food and Drug Administration are phytogenic compounds or derivatives thereof. Aspirin, metformin, morphine, vinblastine, vincristine, quinine, artemisinin, etoposide, teniposide, paclitaxel, and camptothecin are examples of natural compound-derived pharmaceuticals [10]. About 1200 plants have been claimed to contain compounds with antidiabetic properties, and over 400 plants and their bioactive compounds have been scientifically evaluated for type 2 diabetes treatment [11]. However, very little is known about the mechanism of action of plants traditionally used as antidiabetics, preventing them from being used in diabetes care. Recently, more research is being focused on elucidating the mechanism of action of these plants and their active compounds. In this review, we focus on plant-derived compounds and extracts that affect pancreatic beta cell function. The compounds' chemical structures and actions on pancreatic beta cell function in cell culture systems, animal models, and type 2 diabetic patients are also discussed (Figure 2 and Table 1).

## 2. Methods Used for Literature Collection

A literature survey was performed in “PubMed” using the keywords “anti-diabetic activity, beta cell function, beta cell proliferation, and beta cell differentiation” to evaluate the effects of each natural product. To investigate the response of diabetes to natural products, we included any articles describing the effect of natural product-derived compounds on beta cell function using cell culture and diabetic animal models. To evaluate the compounds' effect on humans, we summarized all relevant reviews, such as cohort/case-control studies, randomized clinical trials, controlled clinical trials, and systemic reviews.

## 3. Plant Extracts for the Regulation of Pancreatic Beta Cell Function

### 3.1. *Bidens pilosa* and Polyynes

*Bidens pilosa* (*B. pilosa*) is traditionally used as an antidiabetic herb in various countries.* B. pilosa* contains flavonoids and polyynes; the latter are reported to possess antidiabetic activity [12]. The bioactive compounds identified in* B. pilosa* are 3 polyynes, 3-*β*-D-glucopyranosyl-1-hydroxy-6(*E*)-tetradecene-8,10,12-triyne, 2-*β*-D-glucopyranosyloxy-1-hydroxy-5(*E*)-tridecene-7,9,11-triyne, and 2-*β*-D-glucopyranosyloxy-1-hydroxytrideca-5,7,9,11-tetrayne (cytopiloyne); cytopiloyne showed improved glycemic control over that of the other two polyynes [13]. Cytopiloyne dose-dependently increased insulin mRNA expression and insulin secretion in rat insulinoma RIN-m5F cells, and calcium, diacylglycerol, and protein kinase C*α* were shown to be involved in increased insulin secretion and production [13].

Several studies have indicated that* B. pilosa* could treat type 1 and type 2 diabetes in animals. Nonobese diabetic (NOD) mice treated with cytopiloyne at 25 *μ*g/kg showed normal levels of glucose and insulin after 10 weeks of treatment [14]. Cytopiloyne at 0.5 mg/kg markedly stimulated insulin production in db/db mice compared with the two other polyynes administered at the same concentration. The administration of an ethanol extract of the aerial part of* B. pilosa* (1 g/kg) lowered blood glucose in db/db mice, and treatment with a mixture of two polyynes (3-*β*-D-glucopyranosyl-1-hydroxy-6(*E*)-tetradecene-8,10,12-triyne and 2-*β*-D-glucopyranosyloxy-1-hydroxy-5(*E*)-tridecene-7,9,11-triyne) significantly reduced blood glucose levels [15].

Despite the antidiabetic activities observed in animal models, there are few clinical studies of* B. pilosa* in humans. Recently, Lai et al. demonstrated that treatment with a* B. pilosa* formulation (400 mg/day) for three months reduced fasting blood glucose levels and hemoglobin A1c (HbA1c) in diabetic patients but increased fasting serum insulin in healthy subjects [16]. Moreover, a combination of the* B. pilosa* formulation with antidiabetic drugs (metformin, acarbose, or glibenclamide) achieved a higher glycemic control in diabetic patients than monotherapy. Treatment with the* B. pilosa* formulation significantly increased pancreatic beta cell function of the study participants as shown by the homeostatic model assessment beta (HOMA-*β*) values. Collectively,* B. pilosa* or cytopiloyne derivatives may be potential agents to treat type 2 diabetes by acting on pancreatic beta cells.

### 3.2. *Capsicum annuum* and Capsaicin

Capsaicin is the major compound in* Capsicum annuum*, commonly referred to as red chili pepper. It is widely used as a spice in Asian and Latin American countries [17]. Treatment of RIN-m5F cells with capsaicin (10 pM–10 nM) increased insulin secretion in a dose-dependent manner, and this effect was mediated by the capsaicin-sensitive afferent neuron transient receptor potential vanilloid receptor 1 calcium channel [18]. Administration of capsaicin to Zucker diabetic fatty (ZDF) rats reduced blood glucose levels and increased plasma insulin levels compared with those of control mice [19]. Dietary supplementation of chili pepper powder for two weeks to streptozotocin- (STZ-) induced diabetic rats fed a high-fat diet did not decrease the blood glucose level, but the plasma insulin level was higher in these rats than that in the control group, suggesting that capsaicin possesses an insulinotropic activity rather than hypoglycemic effect [17]. However, the effects of capsaicin in diabetic patients are unknown.

### 3.3. *Carica papaya*

*Carica papaya* (*C. papaya*) belongs to the family Caricaceae. It is cultivated in most of the tropical countries.* C. papaya* is commonly used in traditional medicine for the treatment of various human diseases including diabetes, obesity, and infection. In particular, the leaves of* C. papaya* show antidiabetic actions. Flavonoids, alkaloids, saponins, and tannins are speculated to be the bioactive phytochemicals in* C. papaya*, but the actual active components have not yet been identified. The aqueous extract of* C. papaya* leaves (0.75 g and 1.5 g/100 mL) significantly reduced plasma blood glucose levels, serum cholesterol, and serum triacylglycerol in STZ-induced and alloxan-induced diabetic rats [20]. Histological staining of the pancreatic islets of Langerhans showed that these extracts significantly induced the regeneration of pancreatic beta cells [21]. Little information exists on the antidiabetic effect of* C. papaya* in humans; therefore, future research is required.

### 3.4. *Gymnema sylvestre*

*Gymnema sylvestre* (*G. sylvestre*) has traditionally been used to treat diabetes in India for centuries. Triterpenoid saponins known as gymnemic acids are the main chemical constituents of* G. sylvestre* and are considered to be the active compounds responsible for the antidiabetic effects of the extracts [22].* G. sylvestre* extract is known to stimulate insulin secretion in various pancreatic beta cell lines, such as HIT-T15 (hamster pancreatic beta cell line) and RIN-m5F cells [23]. In addition, treatment of MIN6 (mouse insulinoma cell line) and isolated human islets of Langerhans with Om Santal Adivasi extract (OSA), a high-molecular-weight leaf extract, stimulated insulin secretion [24, 25]. The insulinotropic activity of* G. sylvestre* extract was mediated via permeabilization of the plasma membrane resulting from the high saponin glycoside content of the extract and increased Ca^2+^ influx through voltage-dependent Ca^2+^ channels [23].

Administration of methanol, acetone, or ethanol extracts of* G. sylvestre* leaves (at a dose of 13.4 mg/kg, 20 mg/kg, and 100 mg/kg, resp.) to diabetic animals (Wistar and Sprague-Dawley rats) significantly increased plasma insulin levels concomitant with decreased glucose levels [26–28]. Treatment of diabetic ob/ob mice with an OSA capsule (500 mg/kg) also decreased plasma glucose levels and significantly induced insulin secretion compared with that in control mice [29]. Another study demonstrated that administration of* G. sylvestre* leaves (200 mg/kg) to alloxan-induced diabetic Wistar rats lowered blood glucose levels through the regeneration of pancreatic beta cells [30].

*G. sylvestre* has shown antidiabetic efficacy in clinical trials.* G. sylvestre* leaves lowered blood glucose levels in type 2 diabetes patients by increasing insulin secretion [31]. In a cohort study with type 2 diabetes patients, oral administration of OSA (1 g/day, 60 days) induced significant increases in circulating insulin and C-peptide concomitant with a significant reduction in blood glucose levels [32]. Therefore, the* G. sylvestre* extract showed hypoglycemic effects via the increase in pancreatic beta cell regeneration and insulin secretion.

### 3.5. *Momordica charantia*

Bitter melon, the fruit of the plant* Momordica charantia* (*M. charantia*), is also known as bitter guard, karela, or balsam pear [33]. It is referred to as “vegetable insulin” because its extract components share structural similarities with animal insulin [34]. The fruit and the whole plant are believed to possess antidiabetic properties [35], and the biochemistry and bioactivity underlying the antidiabetic effect of the extracts of* M. charantia* have been extensively studied. Treatment with a water extract of* M. charantia* prevented alloxan-induced pancreatic beta cell apoptosis and increased insulin secretion in HIT-T15 cells [36].

Extracts of the fruit pulp, seeds, leaves, or whole plant of* M. charantia* were shown to have a hypoglycemic effect in diabetic animal models. A daily oral administration of* M. charantia* fruit juice significantly increased pancreatic beta cell numbers compared to untreated diabetic rats [37]. Aqueous, ethanol, or acetone extracts of* M. charantia* showed an antihyperglycemic effect in STZ- or alloxan-induced diabetic rats [38–40], and its seed extract also showed a glucose-lowering effect in diabetic mice [41]. These results suggest that* M. charantia* may either repair damaged pancreatic beta cells or prevent their death.

The results of randomized, double-blind controlled trials and case studies of the hypoglycemic property of* M. charantia* were evaluated, and most of them demonstrated that fasting and postprandial blood glucose levels were significantly reduced by* M. charantia* administration [35, 42, 43]. Although several clinical studies have been performed, their sample sizes were very small. Therefore, clinical trials with sufficient sample size should be performed to evaluate* M. charantia* as a potential treatment for diabetes.

### 3.6. *Nymphaea stellata* and Nymphayol

*Nymphaea stellata* (*N. stellata*), commonly called Egyptian lotus, is a well-known medicinal plant widely used for the treatment of diabetes, inflammation, and liver disorders. The bioactive molecule, nymphayol (25,26-dinorcholest-5-en-3b-ol), a plant sterol, was initially isolated from the chloroform extract of the flower of* N. stellata* [44]. Oral administration of flower and leaf extracts of* N. stellata* lowered blood glucose levels and increased insulin levels in STZ-induced diabetic rats and alloxan-induced Wistar rats [44–46]. Immunostaining of pancreatic sections from nymphayol-treated diabetic rats showed increased numbers of insulin-positive cells in the islets of Langerhans [44], suggesting that stimulation of pancreatic beta cell regeneration and the subsequent release of insulin are one of the potential mechanisms underlying nymphayol's antidiabetic effect. However, the effect of nymphayol in type 2 diabetic patients is largely unknown.

### 3.7. *Panax ginseng* and Ginsenosides

*Panax ginseng* (*P. ginseng*) has received attention for its antidiabetic and antiobesity effects in diabetic patients and in animal models of type 2 diabetes. Ginsenosides from ginseng extracts are known to be responsible for these effects.* P. ginseng* extracts and ginsenosides induced insulin secretion and protected pancreatic beta cells from apoptosis. Ginsenoside Rb1 and Rg1 promoted glucose-stimulated insulin secretion in MIN6 cells [47] and protected RIN-m5F cells from high glucose/cytokine-induced apoptosis via a decrease in nitric oxide (NO) production and the downregulation of Fas and caspase-3 gene expression [48]. Extracts of ginseng root have also been shown to protect against cytokine-induced apoptosis of MIN6 cells [49]. Another study proposed that American ginseng root (25 *μ*g/mL) stimulated insulin production and prevented cytokine-induced apoptosis via regulation of uncoupling protein-2 in INS-1 cells, a rat insulinoma cell line [50].

Extracts from roots, berries, or leaves were found to be effective against type 2 diabetes in rodents. Administration of red or green ginseng berry extract (150 mg/kg) significantly reduced blood glucose levels and improved glucose tolerance in STZ-induced diabetic mice. Moreover, insulin secretion was increased in berry extract-treated mice, possibly due to increased pancreatic beta cell proliferation [51]. In ob/ob and db/db mice, oral administration of ginseng berry extract also reduced blood glucose levels [52, 53]. Ginsenosides from leaves and roots also showed glucose-lowering effects in db/db mice [52, 54].

Clinical studies have demonstrated that ingestion of* P. ginseng* (6 g/day) for 12 weeks improved glycemic control in type 2 diabetes patients [55, 56]. However, one study reported that ginseng had no antidiabetic effect in these specific diabetes patients [57]. Since differences in the concentrations of the various ginsenosides may have been the cause of the outcome variability, standardization of the types of ginsenoside and their ratios are needed to obtain consistent efficacy.

## 4. Natural Bioactive Compounds for the Regulation of Pancreatic Beta Cell Function

### 4.1. Berberine

Berberine is an isoquinoline derivative alkaloid isolated from rhizoma coptidis, which is used to treat diabetes in China [58]. The effect of berberine on insulin secretion is controversial. Chronic treatment with berberine increased insulin secretion in a dose-dependent manner (1–10 *μ*M) in HIT-T15, MIN6, and mouse islets of Langerhans [59, 60]. However, acute treatment with high concentrations (50 *μ*M for 1 h) reduced insulin secretion [60]. These conflicting results might have been largely owing to the different cell types and experimental conditions used. Although controversial effects on insulin secretion* in vitro* were reported, berberine lowered hyperglycemia, improved insulin resistance, and stimulated pancreatic beta cell regeneration in type 2 diabetic animals. Feeding of db/db mice with berberine (380 mg/kg) resulted in weight loss and a significant improvement in glucose tolerance [61]. Daily administration of berberine for four weeks to STZ-induced diabetic rats significantly reduced oral glucose tolerance compared with that in the control group [59].

In a randomized, double-blind, and placebo-controlled trial, decreased fasting and postprandial plasma glucose with body weight reduction were observed in type 2 diabetic patients after three months of treatment with berberine [62]. A meta-analysis study involving 1068 participants showed that berberine* per se* did not have a glucose-lowering effect in type 2 diabetes patients compared with metformin, glipizide, or rosiglitazone treatment [63] but that the combination treatment with antidiabetic agents showed improved glycemic control over that of either treatment alone [63].

### 4.2. Conophylline

Conophylline (CnP) is a vinca alkaloid extracted from the tropical plant* Ervatamia microphylla* (*E. microphylla*).* E. microphylla* is known to mimic the differentiation-inducing activity of activin A [64]. CnP was found to induce the differentiation of pancreatic progenitor cells to insulin-producing cells. Treatment of acinar carcinoma cells (AR42J) with CnP (0.1 mg/mL) induced the expression of neurogenin-3 by activation of p38 mitogen-activated protein kinase [65], and a combination treatment of CnP (0.4 mg/mL) and betacellulin (1 nM) in ductal cells obtained from neonatal rats stimulated their differentiation into insulin-producing cells [66]. Although activin A has shown effects on beta cell differentiation similar to those of CnP, it also induced apoptosis [67]. Therefore, CnP is preferred in clinical applications because of the lack of apoptosis-inducing activity.

CnP is effective in reversing hyperglycemia in diabetic animal models. A subcutaneous injection of 5 mg/kg CnP reduced blood glucose levels and improved glucose tolerance in neonatal STZ-induced diabetic mice. The number of insulin-positive ductal cells and the pancreatic beta cell mass increased after CnP treatment, suggesting a role for CnP in the differentiation and regeneration of pancreatic beta cells* in vivo* [68]. A combination of CnP (2 *μ*g/g) and betacellulin (200 pmol/g) administered for one week reduced glucose tolerance in neonatal STZ-induced diabetic rats [69]. In addition, CnP administration (9 mg/kg, orally) reduced blood glucose levels and increased plasma insulin levels in Goto-Kakizaki rats after four weeks of treatment [70]. However, only little is known about CnP-rich diets and the incidence of diabetes, warranting further studies.

### 4.3. Curcumin

Curcumin is a major constituent of the rhizomatous powder of* Curcuma longa* (*C. longa,* turmeric) and is commonly used as a food product and medicine in Southern Asia [71]. Curcumin showed a stimulatory effect on insulin secretion by the islets of Langerhans [72]. Curcumin pretreatment of pancreatic islets of Langerhans protected the islets against STZ-induced oxidative stress by scavenging of free radicals and significantly increased cell viability and insulin secretion [73]. Oral administration of curcumin or* C. longa* extract (150–300 mg/kg) significantly reduced blood glucose levels in STZ-induced diabetic rats [74, 75]. Daily intake of curcumin for 70 days along with a high-fat diet also showed a glucose-lowering effect in Sprague-Dawley rats [76]. Curcumin treatment for nine months in a prediabetic population resulted in increased pancreatic beta cell function with high HOMA-*β* [77]. These data suggest that curcumin ameliorates type 2 diabetes via regulation of pancreatic beta cell function.

### 4.4. Epigallocatechin-3-Gallate

Epigallocatechin-3-gallate (EGCG) is a polyphenolic bioactive compound found in green tea (*Camellia sinensis*). EGCG is known to be beneficial as a nutritional supplement against various diseases, including diabetes [78, 79]. EGCG protects against cytokine-, reactive oxygen species- (ROS-), and glucose-induced toxicity. EGCG dose-dependently protected against cytokine-induced cell death in RIN-m5F cells. This effect was mediated by the downregulation of inducible NO synthase expression through the inhibition of nuclear factor-*κ*B (NF-*κ*B) activation [80]. EGCG also protected RIN-m5F cells against high glucose-induced impairment of insulin secretion [81]. A diet supplemented with EGCG ingested for seven weeks improved oral glucose tolerance in ZDF rats and db/db mice [82]. However, contradictory results have been reported in one study: when administrated for four days (5 mg/kg/day) to STZ-induced diabetic rats, EGCG impaired insulin secretion stimulated by high glucose loading [83]. Similarly, it was found that treatment of HIT-T15 cells with EGCG (5–100 *μ*M) decreased cell viability and increased apoptotic cell death concomitant with the production of hydrogen peroxide (H_2_O_2_) and ROS [84]. These results suggest that controlling the EGCG concentration is difficult under experimental conditions.

Several studies demonstrated a potential antidiabetic effect of green tea in healthy subjects but found no significant effect in diabetic patients. Tsuneki et al., for example, found that in healthy Japanese subjects acute and high doses of EGCG-concentrated green tea supplement controlled postprandial hyperglycemia, thus potentially reducing the risk for diabetes [85]. However, in a long-term study performed by Mackenzie et al. [86], no hypoglycemic effect was observed in type 2 diabetic adults who consumed green tea extract.

### 4.5. Genistein

Soybean (*Glycine max*) is an important protein source, and soybean isoflavones have been reported to prevent diabetes [87]. Genistein is a major isoflavone present in* Glycine max*. Genistein is known to have several beneficial effects in pancreatic beta cells, such as increased insulin secretion and cell proliferation and the prevention of pancreatic beta cell apoptosis. Genistein treatment increased glucose-stimulated insulin secretion in MIN6 cells and in isolated mouse and rat islets of Langerhans [88]. However, discrepant effects on insulin secretion were observed depending on the concentrations of genistein used: high concentrations (100 *μ*mol/L) of genistein inhibited insulin secretion in isolated rat islets of Langerhans [89], while physiological concentrations (5 *μ*mol/L) potentiated glucose-stimulated insulin secretion in both pancreatic beta cell lines and isolated mouse islets of Langerhans [90]. In addition, although pancreatic beta cell proliferation reduced and apoptosis increased after treatment with high genistein concentrations, proliferation was inhibited at low genistein concentrations. Acute treatment (24 h) with a low concentration (5 *μ*mol/L) of genistein induced proliferation in INS-1 cell and human islets [91]. Moreover, low doses of genistein reduced sodium fluoride-induced pancreatic beta cell apoptosis [92]. The insulin-secreting activity and proliferative effects in pancreatic beta cells and mouse islets of Langerhans required the activation of protein kinase A and extracellular signal regulated kinase (ERK) [90, 93].

Soy protein containing genistein and daidzein suppressed blood glucose levels in NOD mice by increasing plasma insulin levels [94]. Chronic consumption of a genistein-supplemented diet (250 mg/kg) prevented STZ-induced rises in fasting blood glucose and improved glucose tolerance and circulating insulin levels [95]. Administration of genistein at 10 mg/kg for 10 weeks in STZ-induced diabetic mice significantly reduced fasting blood glucose levels [96].

The effect of genistein in type 2 diabetic patients is largely unknown. However, data from a recent human study investigating the effect of genistein administration in postmenopausal women showed that genistein administration at 54 mg/day decreased fasting glucose levels and increased glucose tolerance and insulin sensitivity [97].

### 4.6. Kinsenoside

*Anoectochilus roxburghii* (*A. roxburghii*) is one of the original plants used for diabetes. Kinsenoside is a major constituent isolated from* A. roxburghii*'s n-butanol extract. Kinsenoside exhibited antihyperglycemic activity in STZ-treated rats at dose of 15 mg/kg. More intact pancreatic beta cells were observed in the islets of Langerhans in the kinsenoside-treated group, and glucose tolerance was improved in both diabetic and normal rats [98], suggesting that the hypoglycemic effect could be partially attributed to pancreatic beta cell regeneration. In view of its protective property and hypoglycemic and antioxidant activity, kinsenoside may be a promising candidate as an antidiabetic agent for humans.

### 4.7. Quercetin

Quercetin is a natural polyphenolic flavonoid found in a wide variety of plants, vegetables, and fruits and displays antidiabetic properties* in vivo*. Quercetin has been shown to increase insulin secretion and protect against cell death from apoptotic stimuli. Quercetin treatment (20 *μ*mol/L) potentiated insulin secretion in INS-1 cells exposed to various secretagogues such as glucose, glibenclamide, or KCl [99] and stimulated insulin release via enhanced Ca^2+^ uptake from isolated islet of Langerhans cells [100]. Quercetin treatment protected pancreatic beta cells from H_2_O_2_-induced damage and interleukin 1*β*-induced nitrite production [101].

Quercetin has beneficial effects in animal models of type 1 and type 2 diabetes. Quercetin (15 mg/kg) for three days induced the regeneration of pancreatic islets of Langerhans and increased insulin release in STZ-induced diabetic rats [102]. Rutin (100 mg/kg), a glycosidic form of quercetin, decreased glucose levels and increased insulin levels in STZ-induced diabetic rats after 45 days of treatment [103]. It also lowered fasting and postprandial blood glucose levels in db/db mice (0.08% diet for seven weeks) [104].

Little information exists on the antidiabetic effects of quercetin in humans. Nevertheless, in a randomized, blinded, crossover study, a single oral dose of quercetin (400 mg) effectively suppressed postprandial hyperglycemia in patients with type 2 diabetes [105].

### 4.8. Resveratrol

Resveratrol (3,5,4′-trihydroxystilbene) is a polyphenolic compound found in plants and has anti-inflammatory, antiaging, and antidiabetic effects [106]. Resveratrol shows beneficial effects for the prevention of diabetes and diabetic complications, but its effect on insulin secretion* in vitro* is controversial. Resveratrol's effect on insulin secretion was found to be concentration-dependent and to depend on the cell lines and experimental design used. Although a wide range of resveratrol concentrations (3–100 *μ*mol/L) had no effect on the insulin secretion by RIN-m5F cells [107], resveratrol (10–100 *μ*mol/L) induced insulin secretion in other cell lines (HIT-T15 and INS-1) [108]. Furthermore, resveratrol treatment suppressed cytokine-induced NF-*κ*B activation and, consequently, reduced damage to isolated rat islets of Langerhans cells [109].

Consistent with* in vitro* reports, the effect of resveratrol on insulin secretion differed depending on the animal model used. In normal control mice and rats, resveratrol (3 mg/kg) increased the plasma insulin levels and reduced blood glucose levels [108]. However, in STZ/nicotinamide-treated diabetic mice, resveratrol treatment (0.5 mg/kg) reduced the plasma insulin levels [110].

Most studies in humans demonstrated that resveratrol improved glucose tolerance. A pilot trial in obese insulin-resistant adults showed decreased glucose tolerance after four weeks of treatment (1-2 g/day) [111]. Bhatt et al. conducted a randomized trial with type 2 diabetic subjects and found that the fasting blood glucose, HbA1c, total cholesterol, triglyceride, and low density lipoprotein concentrations were significantly reduced in the resveratrol group (250 mg/day for three months) compared with those in the control group [112].

### 4.9. Silymarin

Silymarin is a flavonoid mixture composed of silybin, silydianin, and silychristin, which are active components of the milk thistle plant (*Silybum marianum*) [113]. Silymarin is antiapoptotic in cytokine-induced MIN6 cell death. Treatment with 50 *μ*g/mL of silymarin reduced cytokine mixture-induced NF-*κ*B-activated NO production; the effect was mediated by ERK-1 and ERK-2 phosphorylation [114]. It has been reported that silymarin rescued pancreatic beta cell function in diabetic animals. Soto et al., for example, demonstrated that silymarin (150 mg/kg) rescued the expression levels of insulin and pancreatic and duodenal homeobox 1 in islets of Langerhans from alloxan-induced diabetic rats [115]. In a pancreatectomy model, silymarin treatment (200 mg/kg) upregulated the expression level of Nkx6.1 and insulin in the pancreas, thereby increasing and decreasing the serum insulin and glucose levels, respectively [116]. Cotreatment of diabetic patients with insulin and silymarin (200 mg/day) reduced the blood glucose levels after three months of treatment [117]. A randomized double-blind clinical trial also demonstrated a beneficial effect of silymarin (200 mg/day) on hyperglycemia as shown by a significant decrease in HbA1c at four months after treatment [118].

## 5. Conclusions

Natural products, such as plant extracts and their bioactive compounds, are attractive drug candidates and more attention must be paid to their potential use in the treatment and prevention of type 2 diabetes. We reviewed plant extracts and plant-derived bioactive compounds with known beneficial effects on pancreatic beta cell function. Some of these compounds show highly promising effects, which indicate that the dietary intake of these compounds may be a promising strategy for diabetes prevention. Additionally, phytochemical-based therapies may be developed as novel pharmacological approaches for the treatment of diabetes or as adjuvants to support existing monotherapies. However, conclusive evidence of the efficacy and safety of phytochemical-based therapies is still limited, and further studies are needed to elucidate their mechanisms of action as antidiabetic agents.

## Figures and Tables

**Table 1:** Biological functions of plants (bioactive compounds) with confirmed antidiabetic properties.

Botanical name	Active compounds	Effect observed	References
*Anoectochilus roxburghii*	Kinsenoside	Increases pancreatic beta cell regeneration	[98]
*Biden pilosa*	3-*β*-D-Glucopyranosyl-1-hydroxy-6(*E*)-tetradecene-8,10,12-triyne 2-*β*-D-Glucopyranosyloxy-1-hydroxy-5(*E*)-tridecene-7,9,11-triyne 2-*β*-D-Glucopyranosyloxy-1-hydroxy-5,7,9,11-tetrayne (cytopiloyne)	Increases insulin production Enhances insulin secretion	[12–16]
*Camellia sinensis*	Epigallocatechin-3-gallate	Enhances insulin secretion Inhibits pancreatic beta cell apoptosis	[78–86]
*Capsicum annuum*	Capsaicin	Enhances insulin secretion	[17–19]
*Carica papaya*	Flavonoids/alkaloids/saponin/tannins	Enhances insulin secretion	[20, 21]
*Curcuma longa*	Curcumin	Enhances insulin secretion	[71–77]
*Ervatamia microphylla*	Conophylline	Induces differentiation into insulin producing cells	[64–70]
*Glycine max*	Genistein	Enhances insulin secretion Inhibits pancreatic beta cell apoptosis	[87–97]
*Gymnema sylvestre*	Gymnemic acids	Enhances insulin secretion	[22–32]
*Momordica charantia*	Momordicin	Increases pancreatic beta cell regeneration	[33–43]
*Nymphaea stellate*	Nymphayol	Enhances insulin secretion	[44–46]
*Panax ginseng*	Ginsenoside	Enhances insulin secretion Increases proliferation	[47–57]
Rhizoma coptidis	Berberine	Enhances insulin secretion	[58–63]
*Silybum marianum*	Silymarin	Inhibits pancreatic beta cell apoptosis	[113–118]
Commonly found in plants	Resveratrol	Inhibits pancreatic beta cell apoptosis	[106–112]
Commonly found in plants	Quercetin	Enhances insulin secretion Inhibits pancreatic beta cell apoptosis	[99–105]

## Abbreviations

NOD: Nonobese diabetic
HbA1c: Hemoglobin A1c
HOMA-*β*: Homeostatic model assessment beta
ZDF: Zucker diabetic fatty
STZ: Streptozotocin
NO: Nitric oxide
ROS: Reactive oxygen species
NF-*κ*B: Nuclear factor-*κ*B
ERK: Extracellular signal regulated kinase
H_2_O_2_: Hydrogen peroxide.
